# Design and Synthesis of 4-flurophthalimides as potential anticonvulsant agents

**Published:** 2018

**Authors:** Maryam Iman, Sina Fakhari, Mohammad Jahanpanah, Nima Naderi, Asghar Davood

**Affiliations:** a *Chemical Injuries Research Center, Systems Biology and Poisonings Institute, Baqiyatallah University of Medical Sciences, Tehran, Iran. *; b *Student Research Committee, School of Pharmacy, Shahid Beheshti University of Medical Sciences, Tehran, Iran. *; c *Department of Toxicology, School of Pharmacy, Shahid Beheshti University of Medical Sciences, Tehran, Iran.*; d *Department of Medicinal Chemistry, Faculty of Pharmacy, Pharmaceutical Sciences Branch, Islamic Azad University, Tehran, Iran.*

**Keywords:** 4-flurophthalimides, Anticonvulsant, MES, PTZ, Isoindole

## Abstract

Anticonvulsant activity of phthalimide was discovered in 2000 by molecular hybridization of thalidomide and ameltolide. In our present research we report some new 4-substituted derivatives of phthalimide with good activity against the tonic and clonic seizures. A series of novel 4-flurophthalimides designed using bioisosteric replacement were synthesized by condensation of 4-flurophthalic anhydride with appropriate arylamines. The purity of these compounds was determined by TLC and the chemical structures were confirmed by IR and ^1^H-NMR spectroscopy. Anticonvulsant activity of prepared compounds was evaluated using MES and PTZ models. Some of the designed compounds significantly protected mice against the PTZ-induced seizure among which, compound **10** with lipophilic and flexible aromatic moiety was more potent than the reference drug phenytoin and was the most potent in this series of phthalimide derivatives. In the MES model, the prepared phthalimide did not show efficient activity. The prepared compounds are active in clonic seizure.

## Introduction

**Figure 1:**
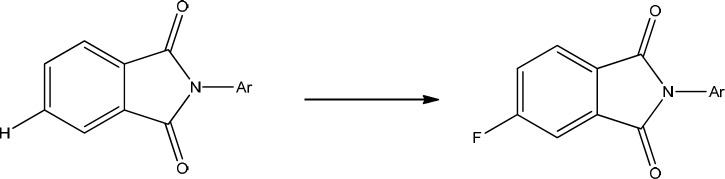
Bioisosteric replacement of hydrogen to design of 4-flurophthalimides

Phthalimide moiety is a multifunctional pharmacophore with cytostatic (1-5), antimicrobial (6-9), anxiolytic (10)**, **hypoglycemic via α-Glucosidase inhibitory (11, 12) analgesic, anti-inflammatory and TNF-α inhibitory (13-17) anti-HIV (18-19) anti-angiogenesis (20, 21) thromboxane inhibitory (22) and anticonvulsant (23-32) activities. Its activity as an anticonvulsant agent was discovered in 2000 by molecular hybridization of thalidomide and ameltolide (28). In our previous research we have reported some new 4-substituted derivatives of phthalimide with good activity against the tonic and clonic seizures (24-26). Our structure activity relationship (SAR) studies revealed that ligands with nitro (NO_2_) and specially amino (NH_2_) moieties at position 4 of phthalimide , due to their ability to create a hydrogen bond with receptor, showed better activity compared to 4-unsubstituted derivatives (24-26). Here in our ongoing research, the synthesis of 4-flurophthalimide derivatives, designed on the base of bioisosteric replacement is reported in which hydrogen atom at the position 4 of phthaslimide was substituted with fluorine atom to achieve the 4-flurophthalimides (Figure 1).

## Experimental

*Chemical synthesis*

**Scheme 1:**
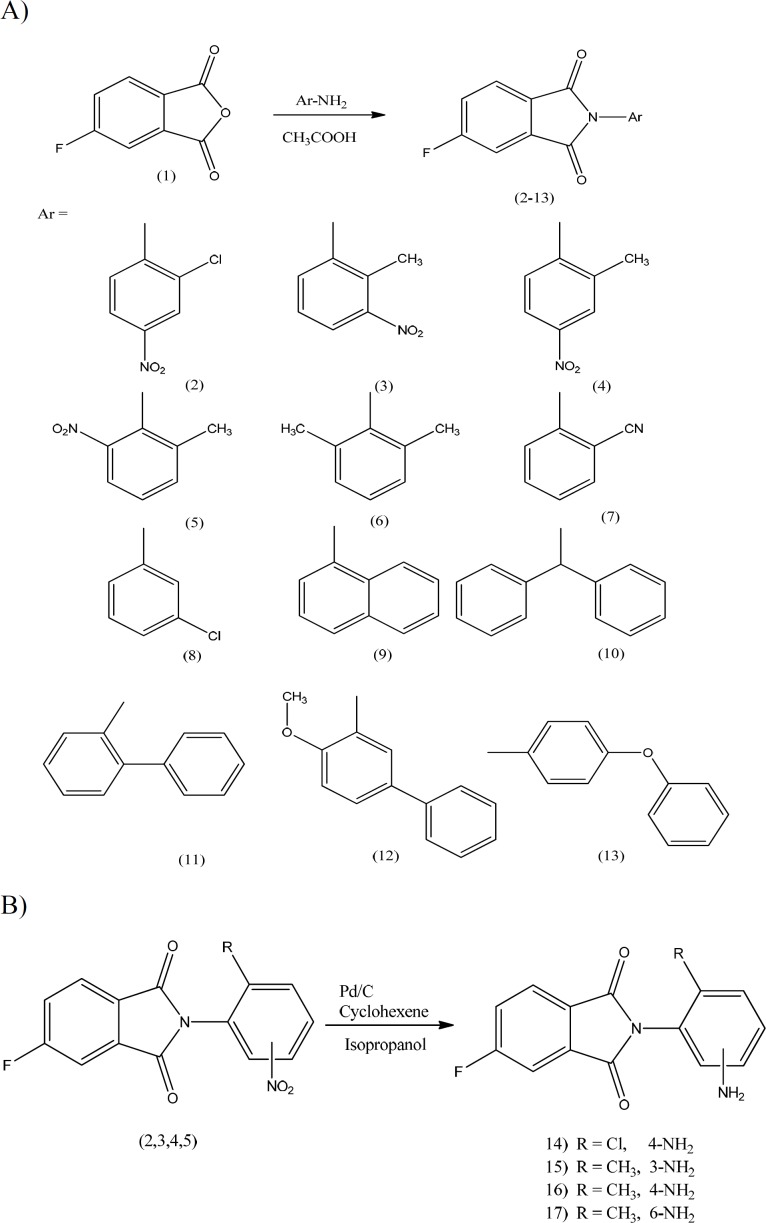
Synthetic route of 4-flurophthalimides 2-17

A group of N-aryl derivatives of the 4-flurophthalimide (compounds 2-13) were synthesized by condensation of the respective aromatic amine with 4-flurophthalic anhydride in acetic acid at reflux temperature (Scheme 1-A). Nitro moiety of compounds 2-5 was reduced to amine using Pd/C and cyclohexene in 2-propanol at reflux temperature (Scheme 1-B) (24-26). All the solvents and chemicals were obtained from Merck and Sigma-Aldrich Company. Thin layer chromatography was performed on plastic TLC plates and TLC spots were visualized using ultraviolet lamp and iodine tank. Melting points were determined using Electro thermal IA 9300 capillary melting-point apparatus (Ontario, Canada). ^1^H-NMR and FT-IR spectra were prepared with the Bruker FT-250 and Nicolet 550-FT spectrometers respectively. A Perkin-Elmer model 240-C apparatus was used for elemental analysis and results were considered in range of + 0.4% of the calculated amounts.

*Preparation of 2-(2-chloro-4-nitrophenyl)-5-fluoro-1H-isoindole-1, 3(2H)-dione (2)*

A solution of 4-flurophthalic anhydride (200 mg, 1.2 mmol) and 2-chloro-4-nitroaniline (184 mg, 1.2 mmol) in glacial acetic acid (2 mL) was stirred and heated under reflux for 120 h. After cooling to room temperature, cold water (5 mL) was added and the resulting precipitate was collected by filtration, washed with cold water and cold ethanol, respectively, and recrystallized from ethanol to give compound 2 as pale yellow crystals (355 mg, 91.9% yield). TLC tank solvent was (dichloromethane/petroleum ether 2:1), m.p. 173-175 °C. ^1^H-NMR (CDCl_3_): δ 7.974 (d, J = 2.5Hz, 1H, H-3-phenyl), 8.299(dd, J = 2.5 and 8.75Hz, 1H, H-5-phenyl), 8.019(d, J=8.51H, H-7-phthalimide), 7.677(dd, J = 2, 2.5 and 6.88Hz, 1H, H-4-phthalimide), 7.573(d, J=8.5Hz, 1H, H-6-phenyl), 7.511 (ddd, J = 1.75, 2.5 and 8.38Hz, 1H, H-6-phthalimide); IR (KBr): ν cm^-^ , 3103 and 3074 (CH-aromatic), 1788 and 1730 (CO), 1526, 1481 and 1375, 1340 (NO_2_). Anal. Cal. For (C_14_H_6_ClFN_2_O_4_): C, 52.44; H, 1.89; N, 8.74; Found: C, 52.49; H, 1.90; N, 8.72.

*Preparation of 5-fluoro-2-(2-methyl-3-nitrophenyl)-1H-isoindole-1, 3(2H)-dione (3)*

Using a procedure similar to that of 2, 4-flurophthalic anhydride and 2-methyl-3-nitroaniline provided the title compound after 96 h that the crude and impure product was recrystallized from ethanol to afford the desired compound as a brown crystals (340 mg, 94%); m.p. 152-153 °C; ^1^H-NMR (CDCl_3_): δ 7.982-8.033(m, 2H, H-7-phthalimide and H-4-phenyl), 7.665(dd, J = 2.25 and 7Hz, H-4-phthalimide), 7.484-7.546(m, 3H, H-6-phthalimide and H-5,6-phenyl), 2.358 ppm (s, 3H, CH_3_); IR (KBr): ν cm^-^ , 3095 and 3076 (CH-aromatic), 2923 (CH-aliphatic), 1778 and 1722 (CO), 1531, 1475 and 1263, 1240 (NO_2_). Anal. Cal. For (C_15_H_9_FN_2_O_4_): C, 60.01; H, 3.02; N, 9.33; Found: C, 60.09; H, 3.03; N, 9.31.

*Preparation of 5-fluoro-2-(2-methyl-4-nitrophenyl)-1H-isoindole-1,3 (2H)-dione (4)*

Using a procedure similar to that of 2, 4-flurophthalic anhydride and 2-methyl-4-nitroaniline provided the title compound after 96 h that the crude and impure product was recrystallized from ethanol to afford the desired compound as a white crystals (350 mg, 96.8%); m.p. 170-171 °C; ^1^H-NMR (CDCl_3_): δ 8.266(s, 1H, H-3-phenyl), 8.205(d, J = 8.5Hz, 1H, H-5-phenyl), 8.003(dd, J = 4.5 and 8 Hz, 1H, H-7-phthalimide), 7.66(dd, J = 2 and 7Hz, H-4-phthalimide), 7.566(t, J = 8Hz, H-4-phenyl), 7.513(dt, J= 2, 2.25 and 8.625 Hz, 1H, H-6-phthalimide), 7.406(d, J = 8.5Hz, 1H, H-6-phenyl), 2.337 ppm (s, 3H, CH_3_). IR (KBr): ν cm^-^ , 3103 and 3076 (CH-aromatic), 2926 (CH-aliphatic), 1780 and 1720 (CO), 1520, 1485 and 1375, 1344 (NO_2_). Anal. Cal. For (C_15_H_9_FN_2_O_4_): C, 60.01; H, 3.02; N, 9.33; Found: C, 60.07; H, 3.03; N, 9.34.

*Preparation of 5-fluoro-2-(2-methyl-6-nitrophenyl)-1H-isoindole-1, 3(2H)-dione (5)*

Using a procedure similar to that of 2, 4-flurophthalic anhydride and 2-methyl-6-nitroaniline provided the title compound after 144 h that the crude and impure product was recrystallized from ethanol to afford the desired compound as a pale brown crystals (340 mg, 94%); m.p. 108-110 °C; ^1^H-NMR (CDCl_3_): δ 8.053(d, J= 8.25 Hz, 1H, H-5-phenyl), 7.988(dd, J = 4.5 and 8.25 Hz, 1H, H-7-phthalimide), 7.632-7.693(m, 2H, H-4-phthalimide and H-3-phenyl), 7.566(t, J = 8Hz, H-4-phenyl), 7.501(dt, J= 2, 2,25 and 8.63 Hz, 1H, H-6-phthalimide), 2.334 ppm (s, 3H, CH_3_). IR (KBr): ν cm^-1^, 3072(CH-aromatic), 2923(CH-aliphatic), 1782 and 1728 (CO), 1531, 1481 and 1267, 1238(NO_2_). Anal. Cal. For (C_15_H_9_FN_2_O_4_): C, 60.01; H, 3.02; N, 9.33; Found: C, 60.08; H, 3.01; N, 9.31.

*Preparation of 2-(2, 6-dimethylphenyl)-5-fluoro-1H-isoindole-1, 3(2H)-dione (6)*

Using a procedure similar to that of 2, 4-flurophthalic anhydride and 2, 6-dimethylnitroaniline provided the title compound after 72 h that the crude and impure product was recrystallized from ethanol to afford the desired compound as a white crystals (120 mg, 74%); m.p. 146.5-148 °C; ^1^H-NMR (CDCl_3_): δ 7.975 (dd, J = 4, 5 and 8Hz, 1H, H-7-phthalimide), 7.644 (d, J =7 Hz, 1H, H-4-phthalimide), 7.478 (t, J = 7 and 7.75, 1H, H-6-phthalimide), 7.284 (t, J = 7Hz, H-4-phenyl), 7.19(d, 2H, H-3,5-phenyl), 2.156 ppm (s, 6H, CH_3_). IR (KBr): ν cm^-1^, 3101, 3074 and 3035 (CH-aromatic), 2932(CH-aliphatic), 1776 and 1720 (CO). Anal. Cal. For (C_16_H_12_FNO_2_): C, 71.37; H, 4.49; N, 5.20; Found: C, 71.31; H, 4.51; N, 5.22.

**Table 1:** The ability of 4-flurophthalimide derivatives (2-17) for the protection against PTZ-induced seizure

**Compound**	**PTZ- score 5** **Seizured/total**	***p*** **-value**
2	4/10	0.0108*
3	4/10	0.0108*
4	6/10	0.0867
5	4/10	0.0108*
6	6/10	0.0867
7	4/10	0.0108*
8	4/10	0.0108*
9	4/10	0.0108*
10	0/10	0.0001***
11	6/10	0.0867
12	6/10	0.0867
13	4/10	0.0108*
14	4/10	0.0108*
15	8/10	0.4737
16	4/10	0.0108*
17	4/10	0.0108*
Phenytoin	2/10	0.0007***
Control (DMSO)	10/10	

* *p *< 0.05,

*** *p *< 0.001 compared with the control group

**Table 2:** The ability of 4-flurophthalimide derivatives (2-17) for the protection against MES-induced seizure.

**Compound**	**MES (protected/total)**
2	2/5
3	2/5
4	0/5
5	1/5
6	1/5
7	0/5
8	0/5
9	1/5
10	1/5
11	1/5
12	1/5
13	0/5
14	2/5
15	2/5
16	2/5
17	0/5
Phenytion	5/5
Control (DMSO)	0/5

*Preparation of 2-(5-fluoro-1,3-dioxo-1,3-dihydro-2H-isoindol-2-yl)benzonitrile (7)*

Using a procedure similar to that of 2, 4-flurophthalic anhydride and 2-aminobenzonitrile provide the title compound after 24 h that the crude and impure product was recrystallized from ethanol to afford the desired compound as a pale yellow crystals (90 mg, 56.2%); m.p. 193.5-196 ºC: ^1^H-NMR (CDCl_3_): δ 8.019(dd, J = 4, 5 and 8.25Hz, 1H, H-7-phthalimide), 7.853(dd, J = 1 and 7 Hz, 1H, H-4-phthalimide), 7.781(dt, J = 1.25, 1.5 and 7.875, 1H, H-6-phthalimide), 7.678(dd, J = 2 and 7Hz, 1H, H-6-phenyl), 7.61(dd, J = 1 and 7.75Hz, 1H, H-3-phenyl), 7.464-7.565 ppm(m, 2H, H-4,5-phenyl). IR (KBr): ν cm^-1^, 3067 (CH-aromatic), 2230(CN), 1788 and 1732 (CO). Anal. Cal. For (C_15_H_7_FN_2_O_2_): C, 67.67; H, 2.65; N, 10.52; Found: C, 67.60; H, 2.64; N, 10.55.

*Preparation of 2-(3-chlorophenyl)-5-fluoro-1H-isoindole-1,3 (2H)-dione (8)*

Using a procedure similar to that of **2**, 4-flurophthalic anhydride and 3-chloroaniline provided the title compound after 72 h that the crude and impure product was recrystallized from ethanol to afford the desired compound as a white crystals (135 mg, 81.3%); m.p. 150.5-151.5 °C: ^1^H-NMR (CDCl_3_): δ 7.981(dd, J= 4, 5 and 8.25Hz, 1H, H-7-phthalimide), 7.643(dd, J= 2, 2,25 and 6.875 Hz, 1H, H-4-phthalimide), 7.35-7.52 ppm (m, 5H, H-6-phthalimide and H-2,4,5,6-phenyl). IR (KBr): ν cm^-1^, 3074 (CH-aromatic), 1778 and 1720(CO). Anal. Cal. For (C_14_H_7_ClFNO_2_): C, 61.00; H, 2.56; N, 5.08; Found: C, 61.09; H, 2.57; N, 5.06.

*Preparation of 5-fluoro-2-(naphthalen-1-yl)-1H-isoindole-1,3 (2H)-dione (9)*

Using a procedure similar to that of 2, 4-flurophthalic anhydride and 1-naphthylamine provided the title compound after 72 h that the crude and impure product was recrystallized from ethanol to afford the desired compound as a violet crystals (150 mg, 85.5%); m.p. 188-191 °C: ^1^H-NMR (CDCl_3_): δ 7.942-8.056(m, 3H, H-7-phthalimide and H-5, 8-naphthyl), 7.697(dd, J = 2, 2.25 and 6.875Hz, 1H, H-4-phthalimide), 7.446-7.641 ppm (m, 6H, H-6-phthalimide and H-2,3,4,6,7-naphthyl). IR (KBr): ν cm^-1^, 3110, 3072 and 3040 (CH-aromatic), 1778 and 1722(CO). Anal. Cal. For (C_18_H_10_FNO_2_): C, 74.22; H, 3.46; N, 4.81; Found: C, 74.18; H, 3.47; N, 4.83.

*Preparation of 2-(diphenylmethyl)-5-fluoro-1H-isoindole-1, 3(2H)-dione (10)*

Using a procedure similar to that of 2, 4-flurophthalic anhydride and 1,1-diphenylmethaneamine provided the title compound after 8 h that the crude and impure product was recrystallized from ethanol to afford the desired compound as a white crystals (185 mg, 92.7%); m.p. 254-256 °C: ^1^H-NMR (DMSO-d6): δ 7.842-7.898(m, 1H, H-7-phthalimide), 7.166-7.7.541(m, 12H, aromatic), 6.290 ppm (s, 1H, CH-N). IR (KBr): ν cm^-1^, 3030 and 3034 (CH-aromatic), 1692 and 1647(CO). Anal. Cal. For (C_21_H_14_FNO_2_): C, 76.12; H, 4.26; N, 4.23; Found: C, 76.19; H, 4.28; N, 4.20.

*Preparation of 2-(biphenyl-2-yl)-5-fluoro-1H-isoindole-1, 3(2H)-dione (11)*

Using a procedure similar to that of 2, 4-flurophthalic anhydride and biphenyl-2-amine provided the title compound after 100 h that the crude and impure product was recrystallized from ethanol to afford the desired compound as a white crystals (150 mg, 78.5%); m.p. 166-168 °C: ^1^H-NMR (CDCl_3_) : δ 7.819(dd, J= 4, 5 and 8.25 Hz, 1H, H-7-phthalimide), 7.46-7.566(m, 5H, H-4-phthalimide and H-2, 5, 3´,5´-biphenyl)), 7.256-7.409 ppm(m, 6H, H-6-phthalimide and H-3,4,2´4´6´-biphenyl). IR (KBr): ν cm^-1^, 3106, 3067 and 3031 (CH-aromatic), 1778 and 1724(CO). Anal. Cal. For (C_20_H_12_FNO_2_): C, 75.70; H, 3.81; N, 4.41; Found: C, 75.65; H, 3.82; N, 4.44.

*Preparation of 5-fluoro-2-(4-methoxybiphenyl-3-yl)-1H-isoindole-1,3 (2H)-dione (12)*

Using a procedure similar to that of 2, 4-flurophthalic anhydride and 4-methoxybiphenyl-3-amine provided the title compound after 72 h that the crude and impure product was recrystallized from ethanol to afford the desired compound as a white crystals (165 mg, 87.9 %); m.p. 201-203 °C: ^1^H-NMR (CDCl_3_) : δ 7.964 (dd, J = 4, 5 and 8.125Hz, 1H, H-7-phthalimide), 7.656(dt, J = 2, 2.5 and 8.375Hz, 1H, H-4-phthalimide), 7.650(s, 1H, H-2-biphenyl), 7.559(d, J = 7Hz, 2H, H-2´,6´-biphenyl), 7.391-7.485(m, 4H, H-6-phthalimide and H-3´,4´,5´-biphenyl), 7.336(d, J = 7.25Hz, 1H, H-6-biphenyl), 7.129(d, J = 8.75Hz, 1H, H-5-biphenyl), 3.845 ppm (3, 3H, OCH_3_). IR (KBr): ν cm^-1^, 3100 and 3057 (CH-aromatic), 1776 and 1722(CO), 1105(OCH_3_). Anal. Cal. For (C_21_H_14_FNO_3_): C, 72.62; H, 4.06; N, 4.03; Found: C, 72.68; H, 4.08; N, 4.06.

*Preparation of 5-fluoro-2-(4-phenoxyphenyl)-1H-isoindole-1, 3(2H)-dione (13)*

Using a procedure similar to that of 2, 4-flurophthalic anhydride and 4-phenoxyaniline provided the title compound after 48 h that the crude and impure product was recrystallized from ethanol to afford the desired compound as a white crystals (190 mg, 94.6%); m.p. 153-154.5 °C: ^1^H-NMR (CDCl_3_): δ 7.968(dd, J = 4, 5 and 8.25Hz, 1H, H-7-phthalimide), 7.633(dd, J = 2 and 7Hz, 1H, H-4-phthalimide), 7.481(dd, J= 2, 2.25 and 6.966 Hz, 1H, H-6-phthalimide) , 7.387-7.434(m, 1H, H-aromatic), 7.370(d, J = 8.75Hz, 4H, H-aromatic), 7.070-7.191 ppm (m, 4H, H-aromatic). IR (KBr): ν cm^-1^, 3105, 3059 and 3034 (CH-aromatic), 1775 and 1709(CO). Anal. Cal. For (C_20_H_12_FNO_3_): C, 72.07; H, 3.63; N, 4.20; Found: C, 72.02; H, 3.62; N, 4.22.

*Preparation of 2-(4-amino-2-chlorophenyl)-5-fluoro-1H-isoindole-1, 3(2H)-dione (14)*

A suspension of compound 2 (200mg, 0.62 mmol), isopropanol (8 mL), cyclohexene (2mL) and Pd/C 10% (45mg, 0.427 mmol) in a 50 mL round-bottomed flask equipped with a reflux condenser was heated and stirred vigorously under reflux for 24 h (scheme 1B). The reaction mixture was filtered and the solvent removed under reduced pressure to get the desire compound as a brown powder (130 mg, 71.7%); TLC (dichloromethane/petroleum ether 2:1); mp 182-184 °C. ^1^H-NMR (CDCl_3_): δ 7.957(dd, J = 4.25 and 8.12Hz, 1H, H-7-phthalimide), 7.62(d,J= 6.25 Hz, 1H, H-4-phthalimide), 7.458(t, J = 7.25 and , 8.5, 1H, H-6-phthalimide), 7.081(d, J = 8Hz, H-6-phenyl), 6.850(s, 1H, H-3-phenyl), 6.675 (d, J= 8Hz, 1H, H-5-phenyl), 3.01 ppm(br, 2H, NH_2_). IR (KBr): ν cm^-1^, 3417 and 3338(NH_2_), 3220 (CH-aromatic), 1780 and 1722 (CO). Anal. Cal. For (C_14_H_8_ClFN_2_O_2_): C, 57.85; H, 2.77; N, 9.64; Found: C, 57.89; H, 2.76; N, 9.62.

*Preparation of 2-(3-amino-2-methylphenyl)-5-fluoro-1H-isoindole-1, 3(2H)-dione (15)*

Using a procedure similar to that of 14, compound 3 (186 mg, 0.62mmol) provided the title compound after 24 h as a light yellow crystals (130 mg, 77.6%); m.p. 219-221 °C: ^1^H-NMR ( CDCl_3_ ): δ 7.966(dd, J= 4.25, 4.5 and 8.125Hz, 1H, H-7-phthalimide), 7.633(dd, J= 2, 2.25 and 6.874 Hz, 1H, H-4-phthalimide), 7.465(dt, J= 2, 2.5, 8.5 and 8.625 Hz, 1H, H-6-phthalimide), 7.211(t, J = 8Hz, H-5-phenyl), 7.012(d, J = 8Hz, 1H, H-6-phenyl), 6.788(d, 

J = 7.25Hz, 1H, H-4-phenyl), 2.053ppm (s, 3H, CH_3_). IR (KBr): ν cm^-1^, 3441 and 3352(NH_2_), 3110 and 3045 (CH-aromatic), 2923 (CH-aliphatic), 1772 and 1706 (CO). Anal. Cal. For (C_15_H_11_FN_2_O_2_): C, 66.66; H, 4.10; N, 10.37; Found: C, 66.71; H, 4.12; N, 10.35. 

*Preparation of 2-(4-amino-2-methylphenyl)-5-fluoro-1H-isoindole-1, 3(2H)-dione (16)*

Using a procedure similar to that of 14, compound 4 (186 mg, 0.62mmol) provided the title compound after 24 h as a light brown crystals (112 mg, 66.9%); m.p. 182.5-184 °C: ^1^H-NMR (CDCl_3_): δ 7.943(dd, J=4.5 and 8Hz, 1H, H-7-phthalimide), 7.613(dd, J = 2 and 6.75 Hz, 1H, H-4-phthalimide), 7.443(dt, J = 2, 2.5 and 8.625, 1H, H-6-phthalimide), 6.959(d, J = 8.25Hz, H-6-phenyl), 6.657(s, 1H, H-3-phenyl), 6.623(d, J = 8.5Hz, 1H, H-5-phenyl), 2.80(brs, 2H, NH_2_), 2.092ppm (s, 3H, CH_3_). IR (KBr): ν cm^-1^, 3439 and 3360(NH_2_), 3097 and 3077 (CH-aromatic), 2966 (CH-aliphatic), 1776 and 1718 (CO). Anal. Cal. For (C_15_H_11_FN_2_O_2_): C, 66.66; H, 4.10; N, 10.37; Found: C, 66.62; H, 4.08; N, 10.39.

*Preparation of 2-(2-amino-6-methylphenyl)-5-fluoro-1H-isoindole-1, 3(2H)-dione (17)*

Using a procedure similar to that of 14, compound 5 (186 mg, 0.62 mmol) provide the title compound after 24 h as a yellow crystals (110 mg, 65.7%); m.p. 153-155 °C: ^1^H-NMR (CDCl_3_): δ 8.31(dd, J = 4.5 and 8.25 Hz, 1H, H-7-phthalimide), 7.930(dd, J = 2.01 and 6.76 Hz, 1H, H-4-phthalimide), 7.439-7.70(m,2H, H-6-phthalimide and H-4-phenyl), 7.065-7.45(m, 2H, H-3,5-phenyl), 2.09 ppm (s, 3H, CH_3_). IR (KBr): ν cm^-1^, 3430 and 3355(NH_2_), 3095 and 3070 (CH-aromatic), 2923(CH-aliphatic), 1761 and 1722, (CO). Anal. Cal. For (C_15_H_11_FN_2_O_2_): C, 66.66; H, 4.10; N, 10.37; Found: C, 66.63; H, 4.11; N, 10.39.

*Pharmacology*

The test compounds were evaluated for anticonvulsant activities using maximal electroshock- (MES) and pentylenetetrazole- (PTZ) induced seizure. Male NMRI mice (20 – 25 g; purchased from Pasture Institute, Tehran, Iran) were used for MES test. For PTZ-induced seizure, male wistar rats (150-200 g) were used. Animals were purchased from Pasture Institute (Tehran, Iran) and were kept in controlled light and temperature condition (12 h/12 h light/dark cycle; 22-25 ºC) with free access to food and tap water. The test compounds were dissolved in DMSO and injected intraperitoneally at the dose of 40 mg/kg 30 min before seizure tests. The different test compound-treated groups were compared with the control group (which received DMSO) or phenytoin-treated group (40 mg/kg) as reference drug. The volume of injected drugs/vehicle was 10 mL/kg in mice and 1 mL/kg in rats. Anticonvulsant evaluation by MES test was performed as described previously (33). Electroshock was induced by applying an alternating current (intensity 40 mA, pulse duration 0.2 s, frequency 50 Hz) through ear clip electrodes by a stimulator (Borj Sanat, Iran). The end-point for seizure occurrence was the observation of hind-limb tonic extension (HLTE) in mice. In PTZ-induced seizure, the occurrence of stage 5 of Racine score (rearing and falling with forelimb clonus) (34) was considered as the endpoint and the antiseizure effect was evaluated by measuring the number (ratio) of seizured animals out of total number of animals in each 

group.

*Statistical Analysis*

The results of PTZ and MES test are presented as seizured/total and protected/total, respectively and the percent protection against seizure between the groups was analyzed by Chi-square test. P values less than 0.05 were considered as statistically significant. 

## Results and Discussion

*Chemistry:*

A series of novel 2, 5-disubstituted phthalimides were synthesized in good yield according to the method that was mentioned previously (24-26). Condensation of 4-flurophthalic anhydride with appropriate arylamines in glacial acetic acid resulted in desired 2, 5-disubstituted phthalimide. Reduction of nitro group to amine was done using Pd/C and cyclohexene as catalyzer and hydrogen donor respectively (25). The purity of these compounds was determined by TLC and the chemical structures were confirmed by IR and ^1^H-NMR spectroscopy.

Existence of absorption bands of amide, nitro, aromatic amine groups in IR spectra approved preparing of our desired compounds. In H-NMR spectroscopy, the proton on the phthalic ring in nitro and amino derivatives are shifted downfield and upfield respectively as compared with 5-unsubstituted compounds.

*Pharmacology*

The ability of the compounds 2-17 to protect rats against PTZ- and MES- induced seizure was evaluated and the results were presented in Tables 1 and 2, respectively. For both MES and PTZ model, the test compounds were evaluated for anticonvulsant activity at the dose of 40 mg/kg and were compared with vehicle (control) and phenytoin (40 mg/kg; as reference drug). As shown in Table 1, compounds 2, 3, 5, 7, 8, 9, 10, 13, 14, 16, 17, and also phenytoin significantly protected rats against PTZ-induce clonic seizure compared with the control group. Compound 10 (seizured/total: 0/10) with lipophilic diphenylmethane moiety was more potent than the reference drug phenytoin (seizured/total: 2/10) and was the most potent in this series of phthalimide derivatives. Due to presence of a lipophilic and flexible phenyl group in this ligand, it is suggested that hydrophobic and charge transfer interactions may be involved in drug-receptor profile. Comparing nitro and amine containing compounds revealed that reduction of nitro to amine moiety in compounds 2 and 5 did not affect the potency. Instead, in compounds 3 and 4, this change in chemical structure decreased and increased drug potency, respectively. Evaluation of the activity of compounds 5, 7, and 8 indicated that the existence of electron withdrawing groups (NO_2_, CN and Cl, respectively) in the phenyl ring increased the potency. Comparison of compounds 9, 10, 11, 12, and 13 which contain two aromatic rings in two different position of phthalimide showed that compound 10 with more lipophilic and routable aromatic ring is more active than others.

Results of MES test (as shown in Table 2) indicated that compounds 4, 7, 8, 13, and 17 were ineffective in this model and compounds 2, 3, 14, and 16 showed moderate antiseizure activity. Although MES results showed a trend of attenuating seizure intensity in mice but none of the tested compounds at the dose of 40mg/kg produced significant protection against seizure compared with the control group.

## Conclusion

Sixteen analogs of 4-flurophthalimid were synthesized, purified, and characterized by thin layer chromatography, IR, elemental analysis and H-NMR. Ability of prepared compounds to protect against PTZ induced seizure and MES were evaluated *in-vivo* in mice. Pharmacological results indicated that all the prepared compounds were active in clonic seizure but in the tonic model did not show any efficient activity.
